# A Systematic Review and Meta-Analysis of Outcomes for Type 1 Diabetes after Bariatric Surgery

**DOI:** 10.1155/2016/6170719

**Published:** 2016-06-08

**Authors:** Alexandra Chow, Noah J. Switzer, Jerry Dang, Xinzhe Shi, Christopher de Gara, Daniel W. Birch, Richdeep S. Gill, Shahzeer Karmali

**Affiliations:** ^1^Faculty of Medicine and Dentistry, University of Alberta, Edmonton, AB, Canada T6G 2R7; ^2^Department of Surgery, University of Alberta, Edmonton, AB, Canada T6G 2B7; ^3^Center for the Advancement of Minimally Invasive Surgery (CAMIS), Royal Alexandra Hospital, Edmonton, AB, Canada T5H 3V9; ^4^Department of Surgery, University of Calgary, Calgary, AB, Canada T2N 2T9

## Abstract

*Background*. The utility of bariatric surgery in type 1 diabetes remains controversial. The aim of the present study is to evaluate glycemic control outcomes in obese patients with type 1 diabetes after bariatric surgery.* Methods*. A comprehensive search of electronic databases was completed. Inclusion criteria included human adult subjects with BMI ≥35 kg/m^2^ and a confirmed diagnosis of type 1 diabetes who underwent a bariatric surgical procedure.* Results*. Thirteen primary studies (86 patients) were included. Subjects had a mean age of 41.16 ± 6.76 years with a mean BMI of 42.50 ± 2.65 kg/m^2^. There was a marked reduction in BMI postoperatively at 12 months and at study endpoint to 29.55 ± 1.76 kg/m^2^ (*P* < 0.00001) and 30.63 ± 2.09 kg/m^2^ (*P* < 0.00001), respectively. Preoperative weighted mean total daily insulin requirement was 98 ± 26 IU/d, which decreased significantly to 36 ± 15 IU/d (*P* < 0.00001) and 42 ± 11 IU/d (*P* < 0.00001) at 12 months and at study endpoint, respectively. An improvement in* HbA1c* was also seen from 8.46 ± 0.78% preoperatively to 7.95 ± 0.55% (*P* = 0.01) and 8.13 ± 0.86% (*P* = 0.03) at 12 months and at study endpoint, respectively.* Conclusion*. Bariatric surgery in patients with type 1 diabetes leads to significant reductions in BMI and improvements in glycemic control.

## 1. Introduction

While type 1 diabetes (T1D) has not traditionally been associated with obesity and metabolic syndrome, recent studies indicate that the prevalence of patients with T1D that are overweight or obese is as high as 50%, with 8% to 40% meeting the diagnostic criteria for metabolic syndrome [1]. The rising incidence of obesity in patients with T1D has been shown to be related to the use of intensive insulin therapy to maintain tight glycemic control [2, 3]. Obesity is a growing problem for patients with T1D given the vicious cycle of obesity-induced insulin resistance necessitating increasing insulin requirements to preserve glycemic targets, which leads to further weight gain.

Although bariatric surgery has been proven to be an effective treatment modality for the improvement and resolution of type 2 diabetes (T2D) in patients who are obese [4, 5], the utility of bariatric surgery for T1D remains controversial. The initial belief that normalization of glucose levels in obese patients with T2D after bariatric surgery was primarily attributed to weight loss effects discouraged the application of bariatric procedures in patients with T1D, since autoimmune destruction of beta cells rather than obesity-induced insulin resistance was considered the major pathophysiologic factor for the development of T1D. While some authors suggest that weight loss independent mechanisms such as gastrointestinal hormone modulation also play a part in the remission and improvement of T2D after bariatric surgery [6, 7], the effectiveness of these changes in the absence of residual beta cell function, as is the case in T1D, remains unclear. Few data is available regarding the impact of bariatric surgery in obese patients with T1D, although early small case reports suggest that bariatric surgery leads to reduction in insulin requirements in addition to improvements in body mass index and other comorbidities [8].

Therefore, given that the incidence of obesity in patients with T1D is increasing and that obesity can significantly complicate glycemic control, it is necessary to define the effects of bariatric surgery in this population. To date, bariatric surgery outcomes in obese patients with T1D have not been quantitatively summarized, although a number of single center experiences and small case series have been published in the last few years. The aim of this systematic review and meta-analysis is to evaluate the effects of bariatric surgery on body mass index (BMI), total daily insulin requirement, and glycated hemoglobin (HbA1c) in obese patients with T1D.

## 2. Methods

### 2.1. Data Sources

A comprehensive search of electronic databases including MEDLINE, EMBASE, SCOPUS, the Cochrane Library, and Web of Science from 1946 to July 2015 was completed. Title searching was restricted to include “type 1 diabetes mellitus” in conjunction with the following keywords/terms: bariatric, gastric bypass, gastric band, and sleeve gastrectomy. A manual search of the reference lists of pertinent articles was also performed to identify any additional relevant studies.

### 2.2. Selection Criteria

Abstracts were screened by two independent reviewers and selected based on the following inclusion criteria: human studies, adult subjects ≥18 years, clinical obesity BMI ≥35 kg/m^2^ at baseline, primary bariatric surgery performed, diagnosis of T1D at baseline confirmed by the presence of pancreatic autoantibodies (islet cell or glutamic acid decarboxylase), absence of C-peptide, documented history of DKA, and/or insulin therapy required from the time of diagnosis. Articles were excluded if they were letters, comments, or published in abstract form only. Full articles for all selected abstracts were screened more thoroughly using the same selection criteria.

### 2.3. Data Extraction

Pertinent data was collected from full-text articles for all selected abstracts by two independent reviewers and discrepancies were resolved by consensus. While there was more than one publication from the same study population, only the most recent findings were included for the analysis. The primary outcome of interest was preoperative and postoperative HbA1c. Secondary outcomes included basic patient demographics, sample size, duration of study follow-up, type(s) of bariatric surgery performed, weight loss outcomes, and other diabetes related outcomes.

### 2.4. Statistical Analysis

Descriptive categorical data were expressed as percentages and continuous data were expressed as weighted mean ± standard deviation (SD) where appropriate. Meta-analysis was used to compare the outcomes of weight loss and glycemic control indicators (HbA1c and total daily insulin requirement) where data was available. The estimated effects were calculated using the latest version of RevMan software provided by the Cochrane website. The random-effects method was applied in our analysis, assuming that the true effect estimates varied among studies. The included studies were then tested for heterogeneity.

## 3. Results

### 3.1. Study Selection and Characteristics

**Figure 1:**
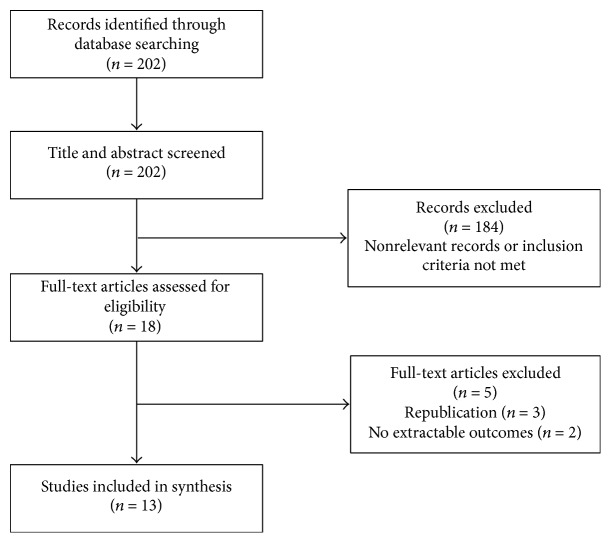
Flow diagram of study selection.

Preliminary searching of the electronic databases identified 202 potentially relevant articles. After screening abstracts against the selection criteria, 18 full-text articles were retrieved and assessed for eligibility. Republication of studies by Czupryniak et al. and Middelbeek et al. was excluded (*n* = 3), as well as studies where the primary outcome of interest could not be extracted (*n* = 2) [9, 10]. In total, 13 primary studies comprising 86 subjects were included in this systematic review and meta-analysis (Figure 1). Included studies were published between February 2010 and July 2015; 11 were case series and 2 were case reports [8, 11–22] (Table 1). Of note, one of the case reports described by Chuang et al. was excluded as the patient was <18 years old and was clinically diagnosed with T2DM at baseline [17]. Unfortunately, no randomized control trials meeting the selection criteria were identified. All studies had clear study designs, aims, consecutive study patients, and appropriate endpoints and follow-up times. Only one study had a follow-up period of less than 12 months [20]. No major methodology flaws were identified.

### 3.2. Baseline Patient Demographics

A total of 86 patients were included in this analysis. Subjects had a mean age of 41.16 ± 6.76 years (*n* = 64) with a preoperative weight and BMI of 123.50 ± 3.84 kg (*n* = 21) and 42.50 ± 2.65 kg/m^2^ (*n* = 86), respectively. Mean duration of diabetes was 22.40 ± 3.81 years (*n* = 54). Roux-en-Y gastric bypass (RYGB) was the favored procedure accounting for 69% (*n* = 59/86) of bariatric surgeries performed, followed by sleeve gastrectomy and biliopancreatic diversion at 15% (*n* = 13/86) and 14% (*n* = 12/86), respectively. Brethauer et al. reported two cases of adjustable gastric banding [11] comprising 2% (*n* = 2/86) of the bariatric operations in this analysis. No mortality was reported in any of the studies.

### 3.3. Meta-Analysis of Weight Loss Outcomes

**Figure 2:**
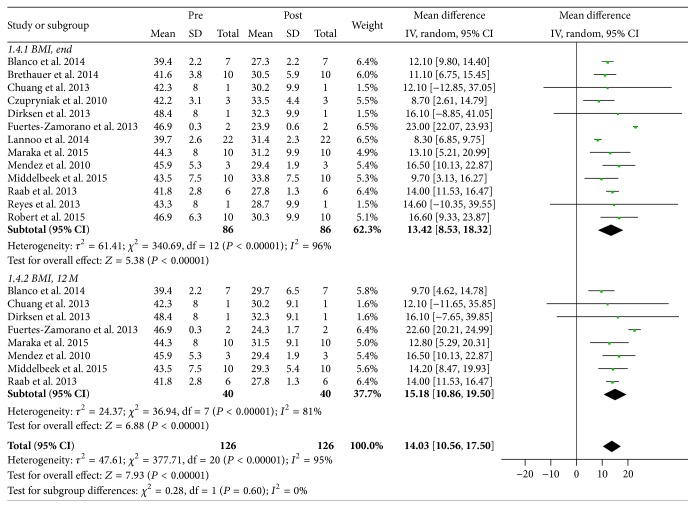
Meta-analysis of changes to BMI postoperatively at 12 months and at study endpoint. End indicates study endpoint; 12 M, at 12 months; pre, preoperative; post, postoperative; CI, confidence interval.

Weight loss outcomes were evaluated based on changes to BMI postoperatively at 12 months and at study endpoint. Eight studies reported BMI at 12 months postoperatively (*n* = 40) [13, 14, 16–19, 21, 22]; BMI at study endpoint was reported for all studies. There was a marked reduction in BMI postoperatively at 12 months and at study endpoint to 29.55 ± 1.76 kg/m^2^ (*P* < 0.00001) and 30.63 ± 2.09 kg/m^2^ (*P* < 0.00001), respectively (Figure 2).

### 3.4. Meta-Analysis of Glycemic Control Outcomes

**Figure 3:**
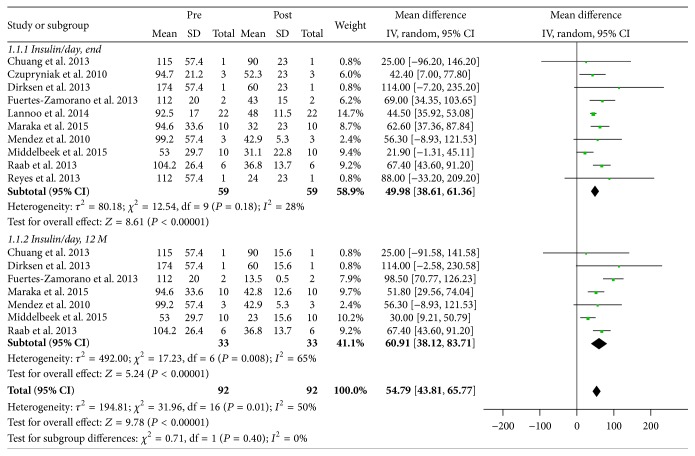
Meta-analysis of changes to total daily insulin requirement postoperatively at 12 months and at study endpoint. End indicates study endpoint; 12 M, at 12 months; pre, preoperative; post, postoperative; CI, confidence interval.

**Figure 4:**
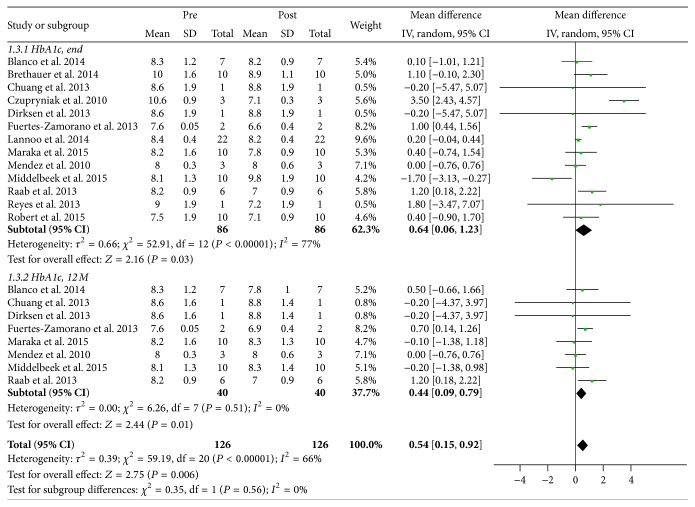
Meta-analysis of changes to HbA1c postoperatively at 12 months and at study endpoint. End indicates study endpoint; 12 M, at 12 months; pre, preoperative; post, postoperative; CI, confidence interval.

Changes to both total daily insulin requirement and HbA1c were analyzed to determine the effect of bariatric surgery on glycemic control. Postoperative daily insulin requirement at 12 months and study endpoint was reported in seven (*n* = 33) [14, 16–19, 21, 22] and ten studies (*n* = 59) [8, 12, 15–22], respectively. Preoperative weighted mean total daily insulin requirement was 98 ± 26 IU/d, which decreased significantly to 36 ± 15 IU/d (*P* < 0.00001) and 42 ± 11 IU/d (*P* < 0.00001) at 12 months and at study endpoint, respectively (Figure 3). Of note, weight-adjusted total daily insulin requirement at baseline and postoperatively at study endpoint was reported in all but one study (*n* = 76) [14], which also decreased appreciably from 0.78 ± 0.20 IU/d/kg to 0.48 ± 0.11 IU/d/kg, respectively (*P* = 0.0001). In terms of HbA1c, this was reported in eight studies at 12 months postoperatively (*n* = 40) [13, 14, 16–19, 21, 22]; HbA1c at study endpoint was reported for all studies. Weighted mean preoperative HbA1c was 8.46 ± 0.78% (*n* = 86), which decreased to 7.95 ± 0.55% (*P* = 0.01) and 8.13 ± 0.86% (*P* = 0.03) at 12 months and at study endpoint, respectively (Figure 4).

## 4. Discussion

This systematic review and meta-analysis demonstrates that obese patients with T1D are able to achieve marked reductions in BMI after bariatric surgery. Moreover, bariatric surgery not only leads to a substantial decrease in total daily insulin requirement but also improves long-term glycemic control as evidenced by a statistically significant reduction in HbA1c postoperatively. While a qualitative summary of the current literature on the topic of bariatric surgery in patients with T1D has previously been published [23], this study is the first meta-analysis of its kind to evaluate weight loss and glycemic status related outcomes in this population.

Bariatric surgery has been proven to lead to cessation or reduction in insulin requirements for obese patients with T2D on insulin therapy [24]. Many authors suggest that this decrease in need for exogenous insulin is associated with the positive effect of weight loss on insulin sensitivity in the liver and peripheral tissues [6, 25]. Similarly, this analysis reports a marked and sustained reduction in BMI for obese patients with T1D after bariatric surgery (weighted mean difference of 13.42 at study endpoint, *P* < 0.00001) that is accompanied by a reduction in total daily insulin requirement (weighted mean difference of 49.98 IU/d at study endpoint, *P* < 0.00001). The observed correlation between weight loss and reduced insulin requirements suggests that although insulin resistance has traditionally only been associated with the pathophysiology of T2D, it may play a role in obese patients with T1D as well. Furthermore, this finding adds validity to the “accelerator hypothesis” that proposes that T1D and T2D, rather than being separate disease processes, belong to the spectrum of a single disorder driven by insulin resistance, which inevitably leads to beta cell loss but at different tempos depending on genetic background [26]. The existence of latent autoimmune diabetes in adults (LADA), which describes patients with the T2D phenotype who possess islet cell antibodies but experience slowly progressive beta cell failure [27, 28], also supports this hypothesis.

This analysis also reports that obese patients with T1D are able to achieve appreciable reductions in HbA1c after bariatric surgery (weighted mean difference of 0.64% at study endpoint, *P* = 0.03). However, while an improvement in postoperative HbA1c was observed, it failed to meet the recommended target of ≤7.0% considered for optimal glycemic control. The reason for this discrepancy is likely multifactorial. Some studies suggest that the elevated postprandial secretion of GLP-1 observed after bariatric surgery may not have as significant glucagonostatic effects as previously believed [13, 16]. More importantly, GLP-1 has been shown in animal models and isolated human islets to enhance beta cell function, including beta cell glucose sensitivity and stimulation of insulin secretion, as well as inducing beta cell neogenesis and proliferation while suppressing apoptosis [6, 16]. These contributions appear to have less of a positive effect on glycemic control in patients with little to no residual beta cell function, as is the case in T1D. This finding is consistent with three case series included in this analysis that demonstrated improved glycemic control reflected in normalization of HbA1c in patients with C-peptide positive T2D requiring insulin therapy but not in those with C-peptide negative T1D [13–15]. Other studies have suggested that ongoing islet cell regeneration is present even in patients with longstanding T1D and that the timing of bariatric surgery is crucial, as GLP-1 mediated effects may help to preserve beta cell mass and prevent progression to total insulin deficiency if surgery is performed early in the course of the disease [6, 15, 16].

Another complicating factor to good glucose control in the obese patients with T1D given their persistent absolute insulin requirement is the high glucose variability that occurs after bariatric surgery, specifically in RYGB, which accounted for 69% of the procedures in this analysis. High and early postprandial hyperglycemic peaks followed by rapid interstitial glucose decreases are observed after RYGB, which may account for the hypoglycemic-like symptoms that have been previously reported in patients with T2D after bariatric surgery [15, 22, 29] and, in the T1D population, can contribute to a mismatch between blood glucose and subcutaneous insulin administration. Use of sleeve gastrectomy may be more suitable for obese patients with T1D as it facilitates a more predictable absorption of carbohydrates and potentially less glycemic variability than what is seen in RYGB [5, 12].

### 4.1. Limitations

The present study highlights the fact that the metabolic benefits of bariatric surgery in obese patients with T1D are not well studied and remain poorly understood. This analysis is limited by the quality of the available studies, the majority of which were case series with a few case reports. Furthermore, there was significant statistical heterogeneity among the studies in the meta-analysis performed, which limits the conclusions that can be drawn from the available data. In addition, all included studies were insufficiently powered for the primary outcome of HbA1c, with only one study describing an experience of more than 10 subjects. Ideally, randomized controlled trials comparing medical management of obesity to bariatric surgery in patients with T1D are needed, similar to the well-established studies published for T2D [30]. In addition, the current study was unable to address rates of improvement and resolution of comorbid conditions due to insufficient reporting or inconsistent definitions used across the studies. Previous studies have reported conflicting findings regarding improvement of diabetes associated complications and cardiovascular disease [16, 22].

In summary, this systematic review and meta-analysis demonstrates that obese patients with T1D can achieve marked reductions in weight as well as improvements in glycemic status reflected in both total daily insulin requirement and HbA1c after bariatric surgery. Ultimately, further research including randomized controlled trials is necessary to confirm these findings.

## Figures and Tables

**Table 1:** Study characteristics.

Author	Year	Patients (*n*)	RYGB (*n*)	BPD (*n*)	SG (*n*)	AGB (*n*)	Study endpoint (mo)
Maraka et al. [14]	2015	10	9	—	1	—	24
Middelbeek et al. [22]	2015	10	10	—	—	—	60
Robert et al. [15]	2015	10	—	7	3	—	55
Brethauer et al. [11]	2014	10	7	—	1	2	37
Lannoo et al. [12]	2014	22	16	—	6	—	38
Blanco et al. [13]	2014	7	7	—	—	—	24
Dirksen et al. [16]	2013	1	1	—	—	—	12
Chuang et al. [17]	2013	1	—	—	1	—	12
Raab et al. [18]	2013	6	2	3	1	—	12
Fuertes-Zamorano et al. [19]	2013	2	—	2	—	—	54
Reyes Garcia et al. [20]	2013	1	1	—	—	—	10
Mendez et al. [21]	2010	3	3	—	—	—	12
Czupryniak et al. [8]	2010	3	3	—	—	—	76

RYGB indicates Roux-en-Y gastric bypass; BPD, biliopancreatic diversion; SG, sleeve gastrectomy; AGB, adjustable gastric banding.
